# Diabetes mellitus in transfemoral transcatheter aortic valve implantation: a propensity matched analysis

**DOI:** 10.1186/s12933-022-01654-x

**Published:** 2022-11-16

**Authors:** Astrid C. van Nieuwkerk, Raquel B. Santos, Roberto Blanco Mata, Didier Tchétché, Fabio S. de Brito, Marco Barbanti, Ran Kornowski, Azeem Latib, Augusto D’Onofrio, Flavio Ribichini, Jan Baan, Juan Oteo-Dominguez, Nicolas Dumonteil, Alexandre Abizaid, Samantha Sartori, Paola D’Errigo, Giuseppe Tarantini, Mattia Lunardi, Katia Orvin, Matteo Pagnesi, Angie Ghattas, Ignacio Amat-Santos, George Dangas, Roxana Mehran, Ronak Delewi

**Affiliations:** 1 Heart Center, Department of Cardiology, Amsterdam Cardiovascular Sciences, Amsterdam UMC, University of Amsterdam, Meibergdreef 9, 1105AZ Amsterdam, Netherlands; 2 Department of Cardiology, Serviço Cardiologia, Centro Hospitalar Universitário do Porto, Largo Prof. Abel Salazar, 4099-001 Porto, Portugal; 3 Cardiología Intervencionista, Hospital Universitario de Cruces, Baracaldo, Vizcaya Spain; 4 Clinique Pasteur, Toulouse, France; 5 Heart Institute, University of São Paulo Medical School, São Paulo, Brazil; 6 Division of Cardiology, Policlinico-Vittorio Emanuele Hospital, University of Catania, Catania CT, Italy; 7 Cardiology Department, Rabin Medical Center, Petach Tikva, Israel; 8 Division of Cardiology, Department of Medicine, University of Cape Town, Cape Town, South Africa; 9 Montefiore Medical Center, Department of Interventional Cardiology, New York, NY USA; 10 Division of Cardiac Surgery, University of Padova, Padova, Italy; 11 Division of Cardiology, Department of Medicine, University of Verona, Verona, Italy; 12 Hospital Universitario Puerta de Hierro-Majadahonda, Calle Manuel de Falla 1, 28222 Majadahonda, Madrid, Spain; 13 The Zena and Michael A. Wiener Cardiovascular Institute, Icahn School of Medicine at Mount Sinai, New York, NY USA; 14 National Centre for Global Health - Instituto Superiore di Sanità, Rome, Italy; 15 Institute of Cardiology, Department of Medical and Surgical specialties, Radiological sciences and Public Health, ASST Spedali Civili, University of Brescia, Brescia, Italy; 16 CIBERCV, Department of Cardiology, Hospital Clínico Universitario, Valladolid, Spain

**Keywords:** Aortic valve stenosis, Transcatheter aortic valve replacement, TAVI, Diabetes mellitus, Insulin, Mortality, Stroke, Bleeding

## Abstract

**Background:**

Diabetes Mellitus (DM) affects a third of patients with symptomatic severe aortic valve stenosis undergoing transcatheter aortic valve implantation (TAVI). DM is a well-known risk factor for cardiac surgery, but its prognostic impact in TAVI patients remains controversial. This study aimed to evaluate outcomes in diabetic patients undergoing TAVI.

**Methods:**

This multicentre registry includes data of > 12,000 patients undergoing transfemoral TAVI. We assessed baseline patient characteristics and clinical outcomes in patients with DM and without DM. Clinical outcomes were defined by the second valve academic research consortium. Propensity score matching was applied to minimize potential confounding.

**Results:**

Of the 11,440 patients included, 31% (n = 3550) had DM and 69% (n = 7890) did not have DM. Diabetic patients were younger but had an overall worse cardiovascular risk profile than non-diabetic patients. All-cause mortality rates were comparable at 30 days (4.5% vs. 4.9%, RR 0.9, 95%CI 0.8–1.1, p = 0.43) and at one year (17.5% vs. 17.4%, RR 1.0, 95%CI 0.9–1.1, p = 0.86) in the unmatched population. Propensity score matching obtained 3281 patient-pairs. Also in the matched population, mortality rates were comparable at 30 days (4.7% vs. 4.3%, RR 1.1, 95%CI 0.9–1.4, p = 0.38) and one year (17.3% vs. 16.2%, RR 1.1, 95%CI 0.9–1.2, p = 0.37). Other clinical outcomes including stroke, major bleeding, myocardial infarction and permanent pacemaker implantation, were comparable between patients with DM and without DM. Insulin treated diabetics (n = 314) showed a trend to higher mortality compared with non-insulin treated diabetics (n = 701, Hazard Ratio 1.5, 95%CI 0.9–2.3, p = 0.08). EuroSCORE II was the most accurate risk score and underestimated 30-day mortality with an observed-expected ratio of 1.15 in DM patients, STS-PROM overestimated actual mortality with a ratio of 0.77 and Logistic EuroSCORE with 0.35.

**Conclusion:**

DM was not associated with mortality during the first year after TAVI. DM patients undergoing TAVI had low rates of mortality and other adverse clinical outcomes, comparable to non-DM TAVI patients. Our results underscore the safety of TAVI treatment in DM patients.

**Trial registration:**

The study is registered at clinicaltrials.gov (NCT03588247).

**Supplementary Information:**

The online version contains supplementary material available at 10.1186/s12933-022-01654-x.

## Background

Diabetes mellitus (DM) is a well-known risk factor associated with worse outcomes in cardiovascular disease and related procedures [1–3]. Diabetic patients undergoing surgical aortic valve replacement (SAVR) have worse short- and long-term outcomes [4]. Transcatheter aortic valve implantation (TAVI) is a percutaneous treatment option for symptomatic severe aortic valve stenosis [5, 6]. DM is common in TAVI patients, present in up to a third of cases [7–9]. DM patients report higher rates of peripheral artery disease, renal failure, and coronary artery disease, which are all associated with increased procedural risk in TAVI [3, 10, 11]. However, how DM influences outcomes after TAVI procedures remains controversial. Some studies describe similar rates of complications [8, 12–14], while others report higher [7, 9] or even lower one-year mortality rates [15, 16]. This controversy is represented in mortality risk prediction scores, which are based on data from SAVR patients. STS-PROM (Society of Thoracic Surgeons Predicted Risk of Mortality) includes diabetes as a risk factor and had the most accurate prediction in non-diabetic TAVI patients [17]. However, Logistic EuroSCORE (European System for Cardiac Operative Risk Evaluation) does not include DM as a risk factor [18], whereas EuroSCORE II only includes insulin treated DM [19]. The predictive accuracy of these risk scores has not been assessed in diabetic TAVI patients. Therefore the aim of this study was to assess procedural and clinical outcomes in DM patients during the first year after transfemoral TAVI in a real-world global patient population.

## Methods

### Study design and patient sample

The CENTER-collaboration is an international patient level database of subjects with severe aortic valve stenosis undergoing transfemoral TAVI with balloon-expandable (Edwards lifesciences Inc., Irvine, California, USA) or self-expandable valves (Medtronic Inc., Minneapolis, Minnesota, USA). Details on study design, eligibility criteria, systematic search methodology and data collection have been reported previously [20]. The study includes data from 4 single center prospective registries, 3 national registries, 2 multicenter prospective registries, and 1 randomised clinical trial selected through a systematic search. The CENTER-study comprises a global patient sample with patients treated in the United States of America, Brazil, Israel, Spain, Italy and France. All collaborators provided a dedicated database with baseline patient characteristics, echocardiographic data, procedural information and long-term follow-up data. Accordingly, a total of 12,381 patients undergoing transfemoral TAVI between 2007 and 2018 were included in the dataset. DM status was known in 11,440 patients (92%), which were included in the current study. The diagnosis of diabetes mellitus was established according to the World Health Organization criteria: fasting plasma glucose ≥ 126 mg/dL; HbA1c ≥ 6.5%; plasma glucose 2 h after oral glucose tolerance test ≥ 200 mg/dL or a random plasma glucose ≥ 200 mg/dL in the presence of symptoms of hyperglycaemia.

### Study outcomes

The primary outcomes of this analysis were 30-day and one-year all-cause mortality in patients with DM compared to patients without DM. Secondary outcomes included rates of in hospital mortality, stroke, myocardial infarction, life-threatening or major bleeding, requirement for permanent pacemaker, new-onset atrial fibrillation and length of hospital stay. All outcomes were defined according to the standardized definitions of the Second Valve Academic Research Consortium (VARC2) [21].

### Statistical analysis

In addition to the complete case analysis, we applied multiple imputation methods to estimate missing data. The imputation protocol included the chain equation method [22] to create ten imputed data sets for the following covariates: age, body mass index, sex, cerebrovascular events, previous myocardial infarction, previous percutaneous coronary intervention, previous coronary artery bypass graft, hypertension, dyslipidaemia, peripheral vascular disease, coronary artery disease, atrial fibrillation, renal failure, mean aortic valve gradient and logistic EuroSCORE. The imputation procedure and subsequent multivariate regression models were performed according to the Rubin’s protocol under the assumption that missing data were missing at random.

We applied propensity score matching to minimize potential confounding. Univariate predictors (p < 0.1) for either 30-day mortality or diabetes status were selected and added to a multivariate logistic regression model. This logistic regression model calculated the propensity score and included 18 variables: age, sex, body mass index, previous cerebrovascular events, previous myocardial infarction, history of percutaneous coronary intervention, history of coronary artery bypass graft, hypertension, hypercholesterolemia, peripheral vascular disease, significant coronary artery disease, atrial fibrillation, renal failure (glomerular filtration rate [GFR] < 30 ml/min/1.73m^2)^, logistic EuroSCORE, mean aortic valve gradient, valve design (balloon or self-expanding), valve generation, and year of procedure. Patients with diabetes were matched to a non-diabetic patient based on the nearest propensity score using the one-to-one nearest neighbour method. Calliper was 0.2 of the logit of the propensity score and there was no replacement. Additional Figure S1 displays the distribution of the propensity score across the matched and unmatched population. Additional Table S1 presents the coefficients and standard errors for the variables included in the matching model.

Baseline values of continuous variables were tested for normal distribution and reported as mean with standard deviation or median with interquartile range (IQR, 25th-75th percentile). Differences between diabetic and non-diabetic patients were tested with independent t-test or Mann-Whitney U test where applicable. Baseline categorical variables were presented as frequencies and percentages and differences between groups were tested using chi-square test.

In both the matched and unmatched population, differences in rates of in-hospital, 30-day and one-year outcomes between diabetic and non-diabetic patients were assessed. Differences were tested using chi-square test. The relative risk (RR) and corresponding asymptotic two-sided 95% confidence interval (CI) were reported. Time to mortality curves and hazard ratios (HR) were established with cox regression analysis. The DM patient population was stratified into subgroups according to treatment status: insulin dependent DM (IDDM) and non-insulin dependent DM (NIDDM). To adjust for potential confounding, we analysed survival in IDDM vs. NIDDM patients with multivariate cox regression. Baseline variables were tested as predictors for mortality. If p < 0.10 in the univariate model, the variable was added to the multivariate model. HR with corresponding 95%CI were reported for mortality. We assessed the accuracy of 30-day mortality risk prediction scores: STS-PROM, Logistic EuroSCORE and EuroSCORE II. Risk scores were calculated at each center before TAVI implantation. We calculated observed-expected ratios for 30-day mortality. An observed-expected ratio of less than 1 indicated overestimation, whilst a ratio of more than 1 indicated underestimation by the risk score. All statistical tests were two-tailed, and a value of p < 0.05 was considered statistically significant. All calculations were generated by SPSS software (version 26.0 for Windows, SPSS, Inc., Chicago, Illinois) and R Studio (version 3.6.1, Vienna, Austria).

## Results

### Patient population

Of the 11,440 patients included in the analysis, 31% (n = 3550) had DM and 69% (n = 7890) did not have DM. Prevalence of DM increased during the years of the study period: 28.1% between 2007 and 2010; 30.2% between 2011 and 2014; and 34.8% between 2015 and 2018 (p < 0.001). Diabetic patients were younger (80 ± 7 vs. 82 ± 7 years, p < 0.001), more frequently men (47% vs. 41%, p < 0.001) and had a higher body mass index (28 ± 5 vs. 26 ± 5 kg/m^2^, p < 0.001) than non-diabetic patients. Overall, they had a worse cardiovascular risk profile, with a higher prevalence of hypertension and dyslipidaemia. They more frequently reported a history of myocardial infarction, percutaneous coronary intervention, coronary artery bypass grafting, cerebrovascular events and peripheral vascular disease. Statins, aspirin and clopidogrel were more frequently used by DM patients. Diabetic patients had a higher predicted 30-day mortality according to STS-PROM (7.0%, IQR 4.1–13.5% vs. 6.1%, IQR 3.9–12.8%, p < 0.001) and EuroSCORE II (4.7%, IQR 2.7–8.2% vs. 3.7%, IQR 2.2-6.0%, p < 0.001). In contrast, the Logistic EuroSCORE, which does not include DM as a risk factor, was similar for both groups (15.5%, IQR 9.5–23.6% vs. 15.0%, IQR 9.5–22.9%, p = 0.05). Table 1 presents an overview of baseline characteristics.

**Table 1:** Baseline patient characteristics of the unmatched population

	Diabetes (n = 3550)	No diabetes (n = 7890)	p-value
**Demographics**			
Age (years)	80 ± 7	82 ± 7	< 0.001
Women	1893 (53%)	4655 (59%)	< 0.001
Body mass index (kg/m^2^)	28 ± 5	26 ± 5	< 0.001
Body surface area (m^2^)	1.79 ± 0.19	1.73 ± 0.19	< 0.001
**Medical history**			
Stroke or TIA	410 (12%)	770 (10%)	0.004
Myocardial infarction	615 (17%)	933 (12%)	< 0.001
Previous PCI	889 (25%)	1514 (19%)	< 0.001
Previous CABG	579 (16%)	852 (11%)	< 0.001
Hypertension	3040 (86%)	5492 (75%)	< 0.001
Dyslipidaemia	2396 (68%)	3878 (49%)	< 0.001
Peripheral vascular disease	640 (18%)	1090 (14%)	< 0.001
Coronary artery disease	1707 (48%)	3025 (38%)	< 0.001
Atrial fibrillation	945 (27%)	2148 (27%)	0.50
Renal failure	467 (13%)	1089 (14%)	0.35
GFR (ml/min/1.73m^2^)	52.4 (38.3–69.9)	52.3 (38.9–68.9)	0.73
NYHA ≥ 3	776 (39%)	2000 (46%)	< 0.001
**Risk scores**			
Logistic EuroSCORE (%)	15.5 (9.5–23.6)	15.0 (9.5–22.9)	0.05
STS-PROM (%)	7.0 (4.1–13.5)	6.1 (3.9–12.8)	< 0.001
EuroSCORE II (%)	4.7 (2.7–8.2)	3.7 (2.2-6.0)	< 0.001
**Medication**			
Statin	480 (73%)	932 (65%)	< 0.001
Aspirin	1090 (68%)	2593 (64%)	0.002
Clopidogrel	473 (47%)	1089 (41%)	< 0.001
Oral anticoagulation	558 (32%)	1291 (33%)	0.33
**Echocardiographic characteristics**			
LVEF (%)	55 ± 14	56 ± 14	0.005
Max gradient (mmHg)	76 ± 22	81 ± 24	< 0.001
Mean gradient (mmHg)	50 ± 17	52 ± 17	< 0.001
Aortic valve area (cm^2^)	0.66 ± 0.19	0.67 ± 0.20	0.33
Stroke volume (mL)	29 ± 8	29 ± 7	0.42
Stroke volume index (mL/m^2^)	16 ± 5	17 ± 4	< 0.001
**Device Type**			
Balloon-expandable valve	1918 (54%)	4191 (53%)	0.37
Third generation valve	1101 (33%)	2038 (28%)	< 0.001

Values are median (interquartile range), n (%), or mean ± standard deviation. TIA: transient ischemic attack; PCI: percutaneous coronary intervention; CABG: coronary artery bypass grafting; GFR: glomerular filtration rate; New York Heart Association functional class; EuroSCORE: European System for Cardiac Operative Risk Evaluation; STS-PROM: Society of Thoracic Surgeons Predicted Risk of Mortality LVEF: Left Ventricular Ejection Fraction. Stroke volume index: as indexed to body surface area

### Clinical outcomes in the unmatched population

Patients with DM and without DM had comparable rates of in hospital mortality (4.5% vs. 4.9%, RR 0.9, 95%CI 0.8–1.1, p = 0.43), stroke (1.7% vs. 2.0%, RR 0.8, 95%CI 0.6–1.1, p = 0.27), myocardial infarction (0.9% vs. 0.7%, RR 1.4, 95%CI 0.9–2.1, p = 0.18), major or life-threatening bleeding (6.1% vs. 6.7%, RR 0.9, 95%CI 0.8–1.1, p = 0.25), and permanent pacemaker implantation (13.3% vs. 13.1%, RR 1.0, 95%CI 0.9–1.1, p = 0.74). Median length of stay was 7 days in both groups (IQR 5–11, p = 0.96). Moreover, 30-day rates of all-cause mortality (5.4% vs. 6.1%, RR 0.9, 95%CI 0.8–1.1, p = 0.19) and stroke (2.3% vs. 2.6%, RR 0.9, 95%CI 0.7–1.2, p = 0.40) did not differ between diabetic and non-diabetic patients. One year mortality (17.5% vs. 17.4%, RR 1.0, 95%CI 0.9–1.1, p = 0.86) and stroke rates (5.0% vs. 5.4%, RR 0.9, 95%CI 0.8–1.2, p = 0.53) were also similar. Table 2 presents outcomes in the unmatched population.

**Table 2:** Clinical outcomes of patients with versus without diabetes mellitus in the unmatched population

	Diabetes (n = 3550)	No diabetes (n = 7890)	RR (95% CI)	p Value
**Procedural**				
Conversion to open heart surgery	21 (0.7%)	55 (0.8%)	0.9 (0.5–1.4)	0.54
Mortality < 72h	137 (1.7%)	51 (1.4%)	1.2 (0.9–1.7)	0.24
**During hospital admission**				
Mortality	157 (4.5%)	380 (4.9%)	0.9 (0.8–1.1)	0.43
Stroke	60 (1.7%)	159 (2.0%)	0.8 (0.6–1.1)	0.27
Myocardial infarction	31 (0.9%)	51 (0.7%)	1.4 (0.9–2.1)	0.18
Major or life-threatening bleeding	198 (6.1%)	480 (6.7%)	0.9 (0.8–1.1)	0.25
New onset atrial fibrillation	76 (8.0%)	180 (6.8%)	1.1 (0.9–1.5)	0.23
Permanent pacemaker implantation	428 (13.3%)	940 (13.1%)	1.0 (0.9–1.1)	0.74
Length of stay (days)	7 (5–11)	7 (5–11)	-	0.96
**At 30 Days**				
Mortality	171 (5.4%)	439 (6.1%)	0.9 (0.8–1.1)	0.19
Stroke	72 (2.3%)	185 (2.6%)	0.9 (0.7–1.2)	0.40
**At one year**				
Mortality	394 (17.5%)	874 (17.4%)	1.0 (0.9–1.1)	0.86
Stroke	114 (5.0%)	274 (5.4%)	0.9 (0.8–1.2)	0.53

Incidence and relative risk of clinical outcomes in diabetic compared with non-diabetic patients. RR: Relative Risk; CI: Confidence Interval

### Baseline characteristics of the propensity matched population

A total of 3281 patient pairs were obtained using propensity score matching. The matched population had a mean age of 80 ± 7 years and 54% was women. GFR was lower in diabetic patients (51.4 ml/min/1.73m^2^, IQR 37.4–69.0, vs. 54.1 ml/min/1.73m^2^, IQR 40.6–71.4, p < 0.001) and aortic valve area was smaller (0.67 ± 0.19 cm^2^ vs. 0.69 ± 0.22 cm^2^, p < 0.001) than in non-diabetic patients. Baseline medical history, cardiovascular medication, echocardiographic characteristics and device types were comparable between patients with and without DM, as presented in Table 3.

**Table 3:** Baseline patient characteristics of the propensity matched population

	Diabetes (n = 3281)	No diabetes (n = 3281)	p-value	SMD
**Demographics**				
Age (years)	80 ± 6	80 ± 8	0.60	0.013
Women	1736 (54%)	1709 (54%)	0.50	0.019
Body mass index (kg/m^2^)	28 ± 5	28 ± 5	0.95	0.002
Body surface area (m^2^)	1.78 ± 0.18	1.78 ± 0.18	0.96	0.001
**Medical history**				
Stroke or TIA	358 (11%)	342 (11%)	0.52	0.028
Myocardial infarction	523 (16%)	538 (17%)	0.61	0.019
Previous PCI	772 (24%)	781 (25%)	0.79	0.009
Previous CABG	486 (15%)	505 (16%)	0.51	0.025
Hypertension	2710 (85%)	2705 (85%)	0.86	0.007
Dyslipidaemia	2102 (66%)	2104 (66%)	0.96	0.002
Peripheral vascular disease	554 (17%)	547 (17%)	0.82	0.009
Coronary artery disease	1478 (46%)	1503 (47%)	0.53	0.017
Atrial fibrillation	834 (26%)	839 (26%)	0.89	0.005
Renal failure	439 (14%)	403 (13%)	0.19	0.054
GFR (ml/min/1.73m^2^)	51.4 (37.4–69.0)	54.1 (40.6–71.4)	< 0.001	0.096
NYHA ≥ 3	751 (40%)	768 (42%)	0.14	0.054
**Risk scores**				
Logistic EuroSCORE (%)	15.5 (9.5–23.0)	14.6 (9.0-23.3)	0.02	0.036
STS-PROM (%)	7.0 (4.1–13.4)	5.5 (3.3–12.0)	< 0.001	0.158
EuroSCORE II (%)	4.6 (2.7–8.2)	3.7 (2.2–6.4)	< 0.001	0.211
**Medication**				
Statin	456 (72%)	482 (72%)	0.73	0.024
Aspirin	895 (67%)	943 (66%)	0.69	0.018
Clopidogrel	457 (47%)	462 (45%)	0.22	0.061
Oral anticoagulation	505 (35%)	546 (36%)	0.39	0.036
**Echocardiographic characteristics**				
LVEF (%)	55 ± 14	56 ± 15	0.65	0.069
Max gradient (mmHg)	76 ± 22	77 ± 23	0.17	0.002
Mean gradient (mmHg)	48 ± 16	48 ± 16	0.95	0.040
Aortic valve area (cm^2^)	0.67 ± 0.19	0.69 ± 0.22	< 0.001	0.118
Stroke volume (mL)	29 ± 8	29 ± 7	0.61	0.052
Stroke volume index (mL/m^2^)	16 ± 5	16 ± 4	0.42	0.081
**Device Type**				
Balloon-expandable valve	1671 (53%)	1661 (52%)	0.80	0.007
Third generation valve	1031 (32%)	1011 (32%)	0.59	0.016

Values are median (interquartile range), n (%), or mean ± standard devation. TIA: transient ischemic attack; PCI: percutaneous coronary intervention; CABG: coronary artery bypass grafting; GFR: glomerular filtration rate; NYHA: New York Heart Association functional class; EuroSCORE: European System for Cardiac Operative Risk Evaluation; LVEF: Left Ventricular Ejection Fraction; STS-PROM: Society of Thoracic Surgeons Predicted Risk of Mortality; SMD: Standardized Mean Difference; Stroke volume index: as indexed to body surface area

### Clinical outcomes in the propensity matched population

**Fig. 1:**
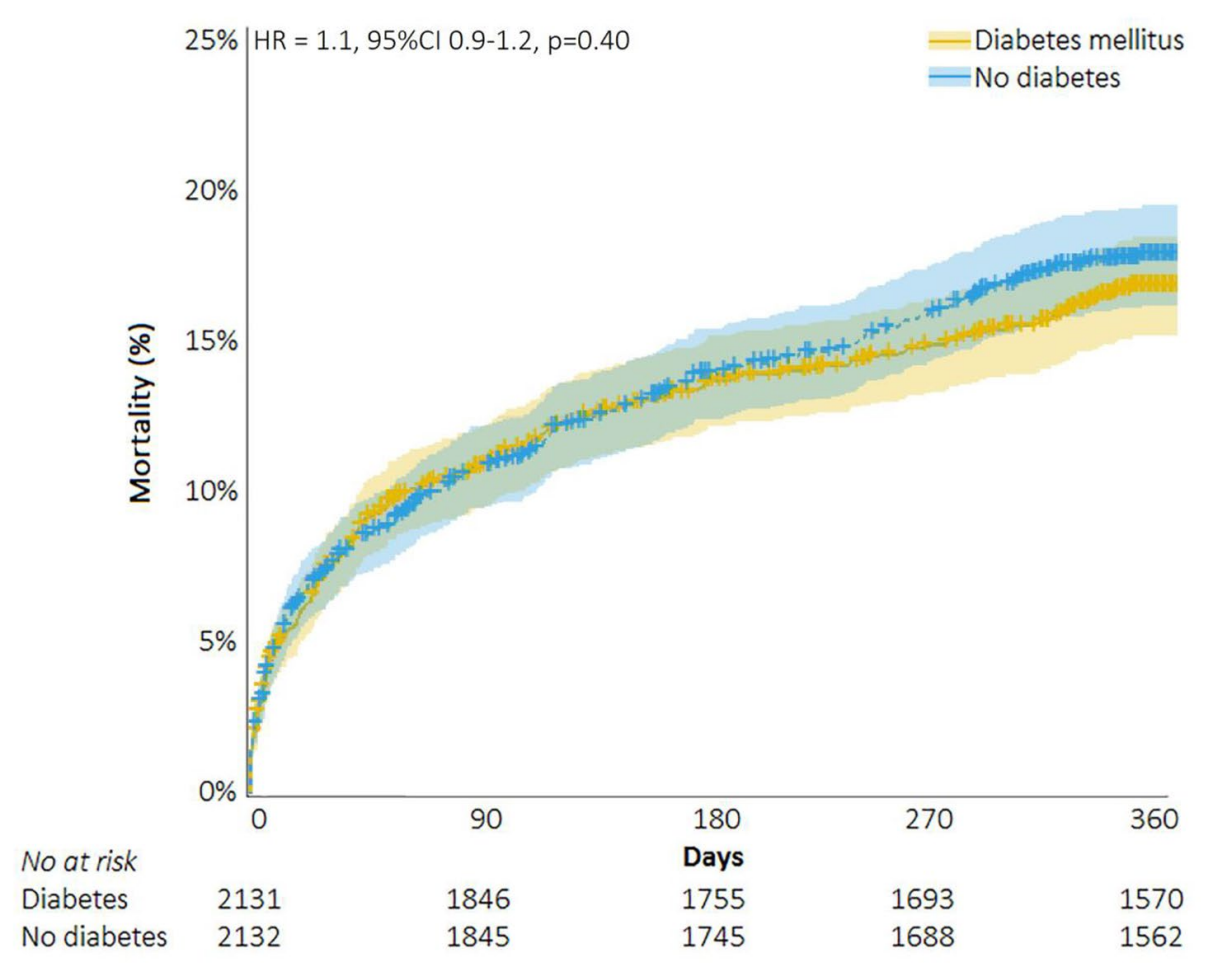
Time-to-mortality curves of patients with and without diabetes mellitus undergoing transcatheter aortic valve implantation (matched population) Legend: HR = Hazard Ratio; CI = Confidence interval.

In hospital outcomes were not different between DM and non-DM patients: mortality (4.7% vs. 4.3%, RR 1.1, 95%CI 0.9–1.4, p = 0.38), stroke (1.7% vs. 2.1%, RR 0.8, 95%CI 0.6–1.2, p = 0.28), myocardial infarction (0.8% vs. 0.5%, RR 1.5, 95%CI 0.8–2.1, p = 0.21), new onset atrial fibrillation (7.8% vs. 7.1%, RR 1.1, 95%CI 0.8–1.6, p = 0.56), and permanent pacemaker implantation (13.0% vs. 13.6%, RR 1.0, 95%CI 0.8–1.1, p = 0.51). Median length of hospital stay was 7 days in both groups (IQR 5–11, p = 0.92). Thirty-day rates of mortality (5.6% vs. 5.6%, RR 1.0, 95%CI 0.8–1.2, p = 0.96) and stroke (2.3% vs. 2.6%, RR 0.9, 95%CI 0.6–1.2, p = 0.47) were comparable, as well as one-year mortality (17.3% vs. 16.2%, RR 1.1, 95%CI 0.9–1.2, p = 0.37) and stroke (4.9% vs. 5.2%, RR 1.0, 95%CI 0.7–1.2, p = 0.75) rates. Figure 1 presents time to mortality curves and Table 4 clinical outcomes in the propensity matched population. DM was not a predictor for one year mortality (HR 1.07, 95%CI 0.92–1.23, p = 0.40): neither in men (HR 0.94, 95%CI 0.76–1.17, p = 0.59) nor in women (HR 1.18, 95%CI 0.67–1.44, p = 0.10).

**Table 4:** Clinical outcomes of patients with versus without diabetes mellitus in the propensity matched population

	Diabetes (n = 3281)	No diabetes (n = 3281)	RR (95% CI)	p Value
**Procedural**				
Conversion to open heart surgery	21 (0.7%)	19 (0.6%)	1.1 (0.6–2.1)	0.73
Mortality < 72h	48 (1.5%)	47 (1.5%)	1.0 (0.7–1.5)	0.92
**During hospital admission**				
Mortality	147 (4.7%)	134 (4.3%)	1.1 (0.9–1.4)	0.38
Stroke	53 (1.7%)	65 (2.1%)	0.8 (0.6–1.2)	0.28
Myocardial infarction	24 (0.8%)	16 (0.5%)	1.5 (0.8–2.1)	0.21
Major or life-threatening bleeding	126 (4.4%)	158 (5.5%)	0.8 (0.6-1.0)	0.05
New onset atrial fibrillation	61 (7.8%)	65 (7.1%)	1.1 (0.8–1.6)	0.56
Permanent pacemaker implantation	400 (13.0%)	418 (13.6%)	1.0 (0.8–1.1)	0.51
Length of stay (days)	7 (5–11)	7 (5–11)	-	0.92
**At 30 Days**				
Mortality	157 (5.6%)	160 (5.6%)	1.0 (0.8–1.2)	0.96
Stroke	65 (2.3%)	75 (2.6%)	0.9 (0.6–1.2)	0.47
**At one year**				
Mortality	370 (17.3%)	349 (16.2%)	1.1 (0.9–1.2)	0.37
Stroke	107 (4.9%)	112 (5.2%)	1.0 (0.7–1.2)	0.75

Incidence and relative risk of clinical outcomes in diabetic compared with non-diabetic patients. The propensity score included: age, sex, body mass index, a history of cerebrovascular events, myocardial infarction, coronary artery bypass graft, percutaneous coronary intervention, hypertension, dyslipidaemia, peripheral vascular disease, atrial fibrillation, renal failure, coronary artery disease, logistic EuroSCORE, mean aortic valve gradient, valve type (self or balloon expanding), valve generation, and year of procedure. RR: Relative Risk; CI: Confidence Interval

### Outcomes in diabetic patients

**Fig. 2:**
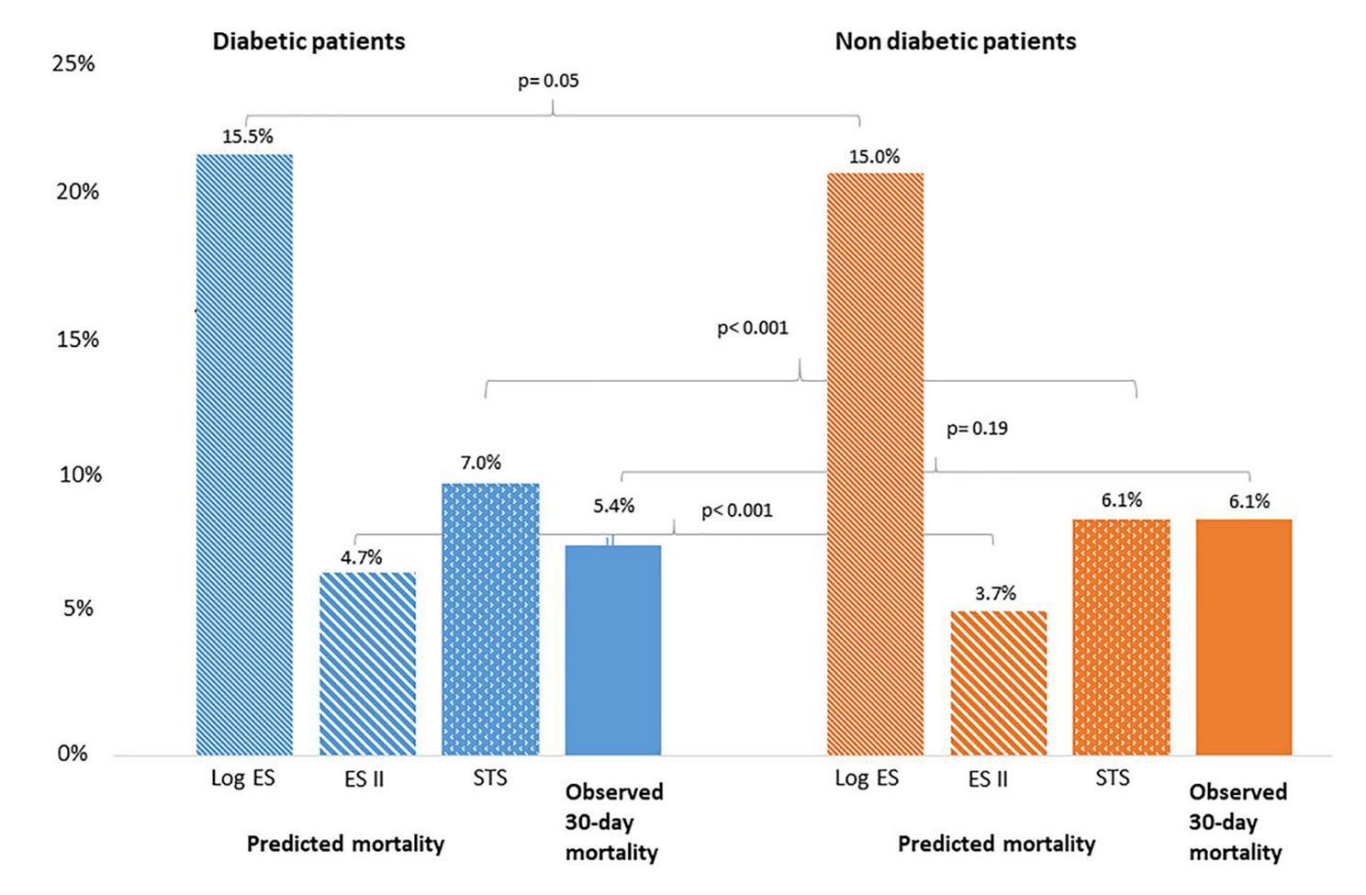
Predicted versus observed mortality in diabetic and non-diabetic patients. Legend: Comparison of predicted mortality (using STS-PROM, Logistic EuroSCORE, and EuroSCORE II) and observed 30-day mortality between diabetic and non-diabetic patients. Log ES = Logistic EuroSCORE; ES II = EuroSCORE II; STS = STS-PROM.

Predicted mortality with STS-PROM, Logistic EuroSCORE and EuroSCORE II was higher in diabetic patients than non-diabetic patients. In DM patients, predicted 30-day mortality with STS-PROM was 7.0%, with Logistic EuroSCORE 15.5% and with EuroSCORE II 4.7%. Observed 30-day mortality in diabetic patients was 5.4%. STS-PROM overestimated 30-day mortality with an observed-expected mortality ratio of 0.77. Logistic EuroSCORE overestimated 30-day mortality with an observed-expected mortality ratio of 0.35. EuroSCORE II underestimated 30-day mortality with a ratio of 1.15. Figure 2 presents an overview of these risk scores.

**Fig. 3:**
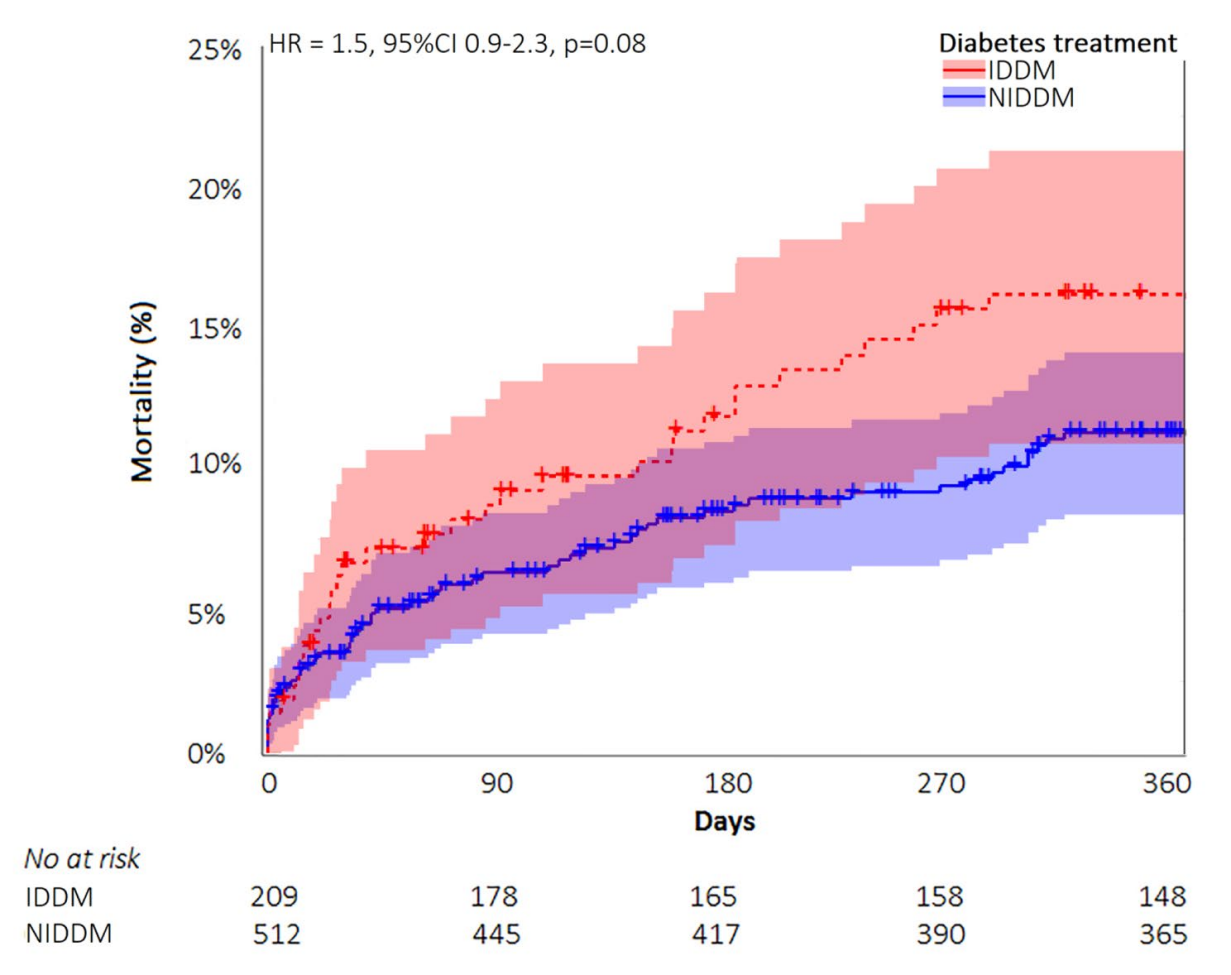
Time-to-mortality curves of patients with insulin dependent versus non-insulin dependent diabetes mellitus undergoing transcatheter aortic valve implantation. Legend: The multivariate model included univariate predictors for mortality: age, body mass index, atrial fibrillation, hypertension, renal failure, and mean aortic valve gradient. IDDM = insulin dependent diabetes mellitus; NIDDM = non-insulin dependent diabetes mellitus; HR = Hazard Ratio; CI = Confidence interval.

In the DM population, independent predictors for 30-day mortality were renal failure (OR 2.1, 95%CI 1.4-3.0, p < 0.001) and baseline atrial fibrillation (OR 2.2, 95%CI 1.6-3.0, p < 0.001). DM patients who had both conditions, were at increased risk for mortality (OR 3.6, 95%CI 2.1–6.1, p < 0.001), compared with patients who had none or only one of these conditions. Diabetes treatment data was available in 1015 of DM patients (29%). IDDM patients (n = 314) were younger, had a higher BMI, and lower GFR than NIDDM patients (n = 701) (Additional Table S2). Table 5 presents clinical outcomes in IDDM and NIDDM patients. There was a trend to higher rates of one-year mortality (15.0% vs. 10.5%, univariate HR 1.5, 95%CI 1.0-2.3, p = 0.08) and stroke (6.5% vs. 3.5%, RR 1.9, 95%CI 0.9–3.9, p = 0.07) in IDDM compared with NIDDM patients. Also in a multivariate model, there was a trend to higher mortality in IDDM, but this did not reach the threshold for statistical significance (HR 1.5, 95%CI 0.9–2.3, p = 0.08). Time to mortality curves for IDDM and NIDDM are depicted in Fig. 3. Rates of other clinical outcomes were similar.

**Table 5:** Clinical outcomes in insulin dependent versus non-insulin dependent diabetic patients

	IDDM (n = 314)	NIDM (n = 701)	RR (95% CI)	P-value
**Procedural**				
Conversion to open heart surgery	1 (0.4%)	3 (0.6%)	0.8 (0.1–7.6)	0.83
**During hospital admission**				
Mortality	8 (2.6%)	14 (2.0%)	1.3 (0.5–3.1)	0.58
Stroke	10 (3.2%)	11 (1.6%)	2.0 (0.9–4.9)	0.10
Myocardial infarction	2 (0.8%)	7 (1.2%)	0.6 (0.1–3.1)	0.57
Major or life-threatening bleeding	40 (13.6%)	86 (13.8%)	1.0 (0.7–1.5)	0.93
New onset atrial fibrillation	9 (6.1%)	15 (4.7%)	1.3 (0.6–3.1)	0.52
Permanent pacemaker implantation	28 (12.3%)	58 (10.9%)	1.1 (0.7–1.9)	0.58
**30 days**				
Mortality	13 (4.4%)	18 (2.7%)	1.6 (0.8–3.4)	0.17
Stroke	11 (3.7%)	12 (1.8%)	2.1 (0.9–4.8)	0.08
**One year**				
Mortality	32 (15.0%)	54 (10.5%)	1.5 (0.9–2.4)	0.09
Stroke	14 (6.5%)	18 (3.5%)	1.9 (0.9–3.9)	0.07

IDDM = insulin dependent diabetes mellitus; NIDDM = non-insulin dependent diabetes mellitus. DM = Diabetes mellitus; RR = Relative Risk; CI = Confidence Interval

## Discussion

### Main results

The main results of this large global propensity matched analysis assessing outcomes in diabetic patients undergoing transfemoral TAVI are: (1) diabetic patients have a worse cardiovascular risk profile and are younger than non-diabetic patients. (2) DM is not associated with worse outcomes within the first year after TAVI. (3) EuroSCORE II was the most accurate risk score with slight underestimation of actual mortality, whereas STS-PROM and logistic EuroSCORE overestimated observed mortality.

### Diabetes as a risk factor for aortic valve stenosis

DM is a major health issue with rising prevalence in the last decades (from 4.7% to 1980 to 8.5% in 2014) [23]. The proportion of DM patients undergoing aortic valve procedures is growing [24–26]. Even during our 11-year study period, we observed a growing percentage of diabetic patients over the years. In fact, DM itself contributes to the pathogenesis of aortic valve stenosis via inflammation and atherosclerosis-like pathways. As a result, aortic valve stenosis is more prevalent in DM patients at a younger age in presence of more comorbidities [24, 26]. In a SAVR study, aortic valve tissue from diabetic patients had increased expression of inflammatory factor NF-κB, which was associated with elevated serum HbA1c levels and increased valvular calcification. Also, valvular expression of coagulation products was higher, especially in poorly controlled diabetes. Thus, elevated serum glucose in DM patients may enhance progression of aortic valve stenosis via these pathways [27].

### Clinical outcomes in DM patients

The results of this propensity matched analysis suggest that clinical outcomes, including mortality rates up to one year after TAVI, are similar in DM and non-DM patients. This is consistent with previous studies [8, 12–14]. However, some earlier studies found increased longer term, but not short term, mortality in DM patients [7, 9]. The question remains whether this increased mortality is due to the direct effect of diabetes on TAVI outcomes, or can be attributed to increased mortality risk of diabetes itself. DM patients in the general population have an excess mortality risk due to increased rates of coronary heart disease, stroke and other vascular diseases [3, 10, 11]. Therefore, it is reassuring that TAVI treatment itself was not associated with increased procedural risk or mortality during the first year. We used propensity score matching to minimize the potential confounding effects of more comorbidities in diabetic patients. *Matsumoto et al.* found higher one-year mortality in DM patients, but did not adjust for confounding factors [7]. The TAVIK registry found higher cross-sectional mortality, but no independent association between DM and 3-year mortality [8]. A sub analysis of the PARTNER trial showed lower mortality in DM patients. They did not correct for confounding: diabetic patients were younger, more frequently men and had a higher BMI. Moreover, these patients were all treated between 2007 and 2009, when patient selection and procedural techniques were different from current practice [15]. In a nationwide 2014–2015 registry in Spain, DM was associated with lower mortality after TAVI in multivariate analysis [16]. However, these results were based on discharge codes, so outcomes completely depend on how well data, including comorbidities, were entered in the administrative database. Nevertheless, not all DM patients share the same negligible procedural risk. DM patients with atrial fibrillation and renal failure were 3.6-fold more likely to die within the first month after TAVI. In the subgroup of DM patients with known treatment status, there was a trend to higher mortality in IDDM patients than NIDDM patients. However, this did not reach statistical significance possibly due to the small portion of IDDM patients. Higher risk patients may benefit from more intensive treatment and closer observation while undergoing TAVI.

### Risk prediction scores

In SAVR patients, DM is a risk factor for unfavourable outcomes [4]. In DM patients treated for severe aortic valve stenosis, TAVI treatment was associated with lower mortality than SAVR treatment [15, 16]. Given the association between DM and post-SAVR mortality, DM has been included in surgical risk prediction scores. The STS-PROM score was developed using a surgical cohort and includes diabetes as a risk factor. STS-PROM was found as best predicting surgical risk score in the overall TAVI patient population [17]. Indeed in our cohort, in non-diabetic patients it precisely predicted 30-day mortality, but it overestimated mortality in diabetic TAVI patients (Fig. 2). Logistic EuroSCORE does not include diabetes as a risk factor [18], but highly overestimated mortality in DM patients. However, Logistic EuroSCORE also overestimated mortality in the general TAVI population [17]. The more recently developed EuroSCORE II includes IDDM as a risk factor [19]. In the current cohort, EuroSCORE II was the most accurate risk score and only marginally underestimated observed 30-day mortality in DM patients. These findings underscore the fact that diabetes itself is not a risk factor for mortality. Furthermore, similar observed mortality despite higher predicted mortality in DM compared with non-DM patients, point toward the fact that DM is not predictive for worse outcomes after TAVI.

### Outcomes according to DM treatment

Insulin treatment has been associated with increased longer term, but not short-term mortality [8, 9]. Also in the current cohort, we observed a trend towards increased rates of mortality and stroke at one year after TAVI in patients with IDDM compared with NIDDM. The diagnosis of diabetes mellitus is based on plasma glucose cut-offs. In fact, DM covers a wide spectrum of more or less severe glucose dysregulation. A more severe dysregulation may affect clinical outcomes more drastically than a prediabetic state. Insulin is used by patients who did not meet adequate glucose control on oral antidiabetics, indicating a more severe diabetic dysregulation. *Chorin et al.* found no differences in mortality according to diabetes treatment (oral or insulin), but instead an association between higher HbA1c levels and increased mortality [14]. IDDM patients may have worse glycaemic control and therefore showed worse clinical outcomes, whilst insulin treatment itself is only a confounder. Moreover, glucose control during the first days after TAVI is important and early hyperglycaemias have been associated with more major complications [28].

### Limitations

Although the included studies were selected through a systematic search, the final study population may be influenced by selection bias for enrolment in each study and for participation of principal investigators. We only included patients that underwent transfemoral TAVI with the two most widely available devices and therefore findings may not be generalizable to non-transfemoral approach and other valve types. Outcomes in non-transfemoral approach should be studied in future trials. Also, this pooled dataset included various study designs without central adjudication of clinical events and therefore event rates may differ between study types. Pulmonary function was not available. Detailed information about the complexity of the disease, such as HbA1c, time since DM diagnosis, dietary treatment and number of medications were not available and how these factors affect outcomes remain unknown. We studied outcomes of TAVI in DM patients up to one year after the procedure, but longer term follow-up including rehospitalisation rates are lacking. Moreover, detailed information about DM treatment including dietary treatment is lacking and should be assessed in future research.

## Conclusion

Diabetic patients undergoing transfemoral TAVI had more cardiovascular comorbidities, were younger and had a higher body mass index than non-diabetic patients. In this large propensity matched analysis, diabetic patients with severe aortic valve stenosis undergoing transfemoral TAVI did not have impaired short-term and one-year clinical outcomes. DM patients had low rates of mortality and other adverse clinical outcomes, comparable to non-DM TAVI patients. In diabetic patients, EuroSCORE II was the most accurate surgical risk score for prediction of 30-day mortality. Our results underscore the safety of TAVI treatment in DM patients.

## Electronic supplementary material

Below is the link to the electronic supplementary material.

**Additional Figure S1. Distribution of the propensity scores in the unmatched and propensity matched population.**
**Additional Table S1. Coefficients for the propensity score matching model.**
**Additional Table S2. Baseline characteristics of patients with insulin dependent versus non-insulin dependent diabetes mellitus.**

## Data Availability

The datasets generated and/or analysed during the current study are not publicly available due to shared copyright of sensitive data but are available from the corresponding author on reasonable request.

## Abbreviations

CENTER: cerebrovascular events in patients undergoing transcatheter aortic valve implantation
CI: confidence interval
EuroSCORE: european system of cardiac operation risk of mortality
HR: hazard ratio
IDDM: insulin dependent diabetes mellitus
IQR: interquartile range
LVEF: left ventricular ejection fraction
NIDDM: non - insulin dependent diabetes mellitus
RR: relative risk
SAVR: surgical aortic valve replacement
STS PROM: society of thoracic surgeons predicted risk of mortality
TAVI: transcatheter aortic valve implantation
VARC2: second valve academic research consortium
