# Supplementary material for: Diabetes mellitus in transfemoral transcatheter aortic valve implantation: a propensity matched analysis
Source: Cardiovasc Diabetol. 2022 Nov 16;21:246. doi: 10.1186/s12933-022-01654-x (PMC9670618; doi:10.1186/s12933-022-01654-x)
Supplement: Supplementary file 1 — Additional Figure S1. Distribution of the propensity scores in the unmatched and propensity matched population. Additional Table S1. Coefficients for the propensity score matching model. Additional Table S2. Baseline characteristics of patients with insulin dependent versus non-insulin dependent diabetes mellitus. [file 12933_2022_1654_MOESM1_ESM.docx]

**Additional Figure S1. Distribution of the propensity scores in the unmatched and propensity matched population

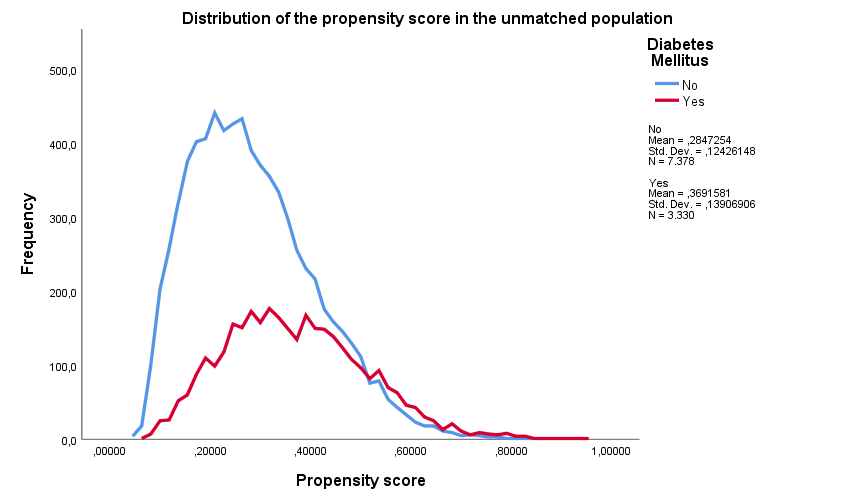
**

**
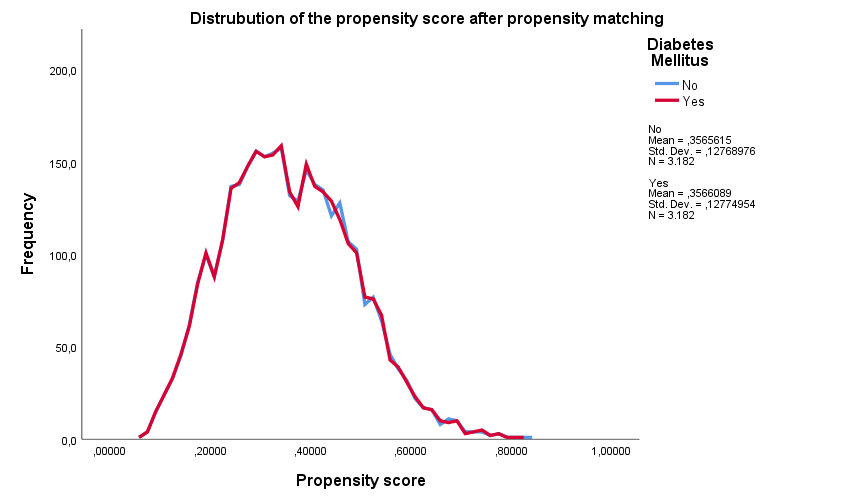
**

**Additional Table S1. Coefficients for the propensity score matching model**

| **Variable** | **Coefficient** | **Standard error** |
| --- | --- | --- |
| Age (per year) | -0.37 | 0.003 |
| Sex (1=male) | 0.113 | 0.046 |
| BMI (per kg/m^2^) | 0.065 | 0.005 |
| Previous cerebrovascular events | 0.084 | 0.071 |
| Previous myocardial infarction | 0.143 | 0.068 |
| Previous coronary artery bypass graft | -0.046 | 0.072 |
| Previous percutaneous coronary intervention | -0.026 | 0.061 |
| Hypertension | 0.462 | 0.060 |
| Dyslipidaemia | 0.550 | 0.047 |
| Peripheral vascular disease | 0.085 | 0.063 |
| Significant coronary artery disease | 0.255 | 0.053 |
| Atrial fibrillation | -0.025 | 0.050 |
| Renal failure (eGFR <30 ml/min/1.73m^2^) | 0.061 | 0.065 |
| Mean aortic valve gradient (per mmHg) | -0.004 | 0.001 |
| Year of procedure | 0.037 | 0.015 |
| Valve design (0=self-expandable, 1= balloon-expandable) | 0.031 | 0.044 |
| Third generation valve | 0.010 | 0.074 |

**Additional** **Table S2. Baseline characteristics of patients with insulin dependent versus non-insulin dependent diabetes mellitus**

|  | **IDDM (n=314)** | **NIDDM (n=701)** | **p-value** |
| --- | --- | --- | --- |
| **Demographics**  Age (years)  Women  Body mass index (kg/m^2^) | 79 ± 8  194 (62%)  29 ±6 | 81 ± 7  419 (60%)  28 ± 5 | 0.004  0.55  0.06 |
| **Medical history**  Stroke or TIA  Myocardial infarction  Previous PCI  Previous CABG  Hypertension  Dyslipidaemia  Peripheral vascular disease  Coronary artery disease  Atrial fibrillation  Renal failure  GFR (ml/min/1.73m^2^) | 43 (14%)  58 (19%)  97 (31%)  71 (23%)  272 (87%)  222 (71%)  69 (22%)  164 (52%)  93 (30%)  54 (17%)  47.9 (35.2-65.1) | 92 (13%)  105 (15%)  170 (24%)  125 (18%)  609 (87%)  487 (69%)  135 (19%)  330 (47%)  206 (29%)  80 (11%)  57.0 (43.6-74.3) | 0.81  0.16  0.03  0.08  0.91  0.69  0.32  0.13  0.94  0.01  <0.001 |
| **Risk scores**  Logistic EuroSCORE (%)  STS-PROM (%)  EuroSCORE II (%) | 17.7 (9.5-26.8)  8.0 (5.0-12.5)  6.9 (4.6-12.3) | 15.6 (10.1-22.8)  5.6 (3.5-8.7)  5.3 (3.0-8.9) | 0.04  <0.001  0.001 |
| **Echocardiographic characteristics**  Max gradient (mmHg)  Mean gradient (mmHg)  Aortic valve area (cm^2^) | 74 ± 21  55 ± 20  0.66 ± 0.19 | 77 ± 22  54 ±19  0.66 ± 0.19 | 0.20  0.25  0.73 |
| **Device Type**  Balloon-expandable valve  Third generation valve | 153 (49%)  117 (50%) | 344 (49%)  311 (56%) | 0.92  0.12 |

*Values are median (interquartile range), n (%), or mean ± standard deviation. TIA: transient ischemic attack; PCI: percutaneous coronary intervention; CABG: coronary artery bypass grafting; GFR: glomerular filtration rate; EuroSCORE: European System for Cardiac Operative Risk Evaluation; STS-PROM: Society of Thoracic Surgeons Predicted Risk of Mortality.*
